# Integrating qualitative research within a clinical trials unit: developing strategies and understanding their implementation in contexts

**DOI:** 10.1186/s13063-024-08124-7

**Published:** 2024-05-16

**Authors:** Jeremy Segrott, Sue Channon, Amy Lloyd, Eleni Glarou, Josie Henley, Jacqueline Hughes, Nina Jacob, Sarah Milosevic, Yvonne Moriarty, Bethan Pell, Mike Robling, Heather Strange, Julia Townson, Qualitative Research Group, C. Drew, D. Gillespie, R. Hale, J. Latchem-Hastings, R. Milton, B. Pell, H. Prout, V. Shepherd, K. Smallman, H. Stanton, Lucy Brookes-Howell

**Affiliations:** 1 Centre for Trials Research, DECIPHer Centre, Cardiff University, Neuadd Meirionnydd, Heath Park, Cardiff, CF14 4YS UK; 2 Centre for Trials Research, Cardiff University, Neuadd Meirionnydd, Heath Park, Cardiff, CF14 4YS UK; 3 Division of Population Medicine, School of Medicine, Cardiff University, Neuadd Meirionnydd, Heath Park, Cardiff, CF14 4YS UK; 4 Wales Centre for Public Policy, Cardiff University, Sbarc I Spark, Maindy Road, Cardiff, CF24 4HQ UK; 5 School of Social Sciences, Cardiff University, King Edward VII Avenue, Cardiff, CF10 3WA UK; 6 DECIPHer Centre, School of Social Sciences, Cardiff University, Sbarc I Spark, Maindy Road, Cardiff, CF24 4HQ UK

**Keywords:** Qualitative research, Qualitative methods, Trials units, Normalisation Process Theory, NPT, Randomised controlled trials, RCTs

## Abstract

**Background/aims:**

The value of using qualitative methods within clinical trials is widely recognised. How qualitative research is integrated within trials units to achieve this is less clear. This paper describes the process through which qualitative research has been integrated within Cardiff University’s Centre for Trials Research (CTR) in Wales, UK. We highlight facilitators of, and challenges to, integration.

**Methods:**

We held group discussions on the work of the Qualitative Research Group (QRG) within CTR. The content of these discussions, materials for a presentation in CTR, and documents relating to the development of the QRG were interpreted at a workshop attended by group members. Normalisation Process Theory (NPT) was used to structure analysis. A writing group prepared a document for input from members of CTR, forming the basis of this paper.

**Results:**

Actions to integrate qualitative research comprised: its inclusion in Centre strategies; formation of a QRG with dedicated funding/roles; embedding of qualitative research within operating systems; capacity building/training; monitoring opportunities to include qualitative methods in studies; maximising the quality of qualitative research and developing methodological innovation. Facilitators of these actions included: the influence of the broader methodological landscape within trial/study design and its promotion of the value of qualitative research; and close physical proximity of CTR qualitative staff/students allowing sharing of methodological approaches. Introduction of innovative qualitative methods generated interest among other staff groups. Challenges included: pressure to under-resource qualitative components of research, preference for a statistical stance historically in some research areas and funding structures, and difficulties faced by qualitative researchers carving out individual academic profiles when working across trials/studies.

**Conclusions:**

Given that CTUs are pivotal to the design and conduct of RCTs and related study types across multiple disciplines, integrating qualitative research into trials units is crucial if its contribution is to be fully realised. We have made explicit one trials unit’s experience of embedding qualitative research and present this to open dialogue on ways to operationalise and optimise qualitative research in trials. NPT provides a valuable framework with which to theorise these processes, including the importance of sense-making and legitimisation when introducing new practices within organisations.

**Supplementary Information:**

The online version contains supplementary material available at 10.1186/s13063-024-08124-7.

## Background

The value of using qualitative methods within randomised control trials (RCTs) is widely recognised [1–3]. Qualitative research generates important evidence on factors affecting trial recruitment/retention [4] and implementation, aiding interpretation of quantitative data [5]. Though RCTs have traditionally been viewed as sitting within a positivist paradigm, recent methodological innovations have developed new trial designs that draw explicitly on both quantitative and qualitative methods. For instance, in the field of complex public health interventions, realist RCTs seek to understand the mechanisms through which interventions generate hypothesised impacts, and how interactions across different implementation contexts form part of these mechanisms. Proponents of realist RCTs—which integrate experimental and realist paradigms—highlight the importance of using quantitative *and* qualitative methods to fully realise these aims and to generate an understanding of intervention mechanisms and how context shapes them [6].

A need for guidance on how to conduct good quality qualitative research is being addressed, particularly in relation to feasibility studies for RCTs [7] and process evaluations embedded within trials of complex interventions [5]. There is also guidance on the conduct of qualitative research within trials at different points in the research cycle, including development, conduct and reporting [8, 9].

A high proportion of trials are based within or involve clinical trials units (CTUs). In the UK the UKCRC Registered CTU Network describes them as:

… specialist units which have been set up with a specific remit to design, conduct, analyse and publish clinical trials and other well-designed studies. They have the capability to provide specialist expert statistical, epidemiological, and other methodological advice and coordination to undertake successful clinical trials. In addition, most CTUs will have expertise in the coordination of trials involving investigational medicinal products which must be conducted in compliance with the UK Regulations governing the conduct of clinical trials resulting from the EU Directive for Clinical Trials.

Thus, CTUs provide the specialist methodological expertise needed for the conduct of trials, and in the case of trials of investigational medicinal products, their involvement may be mandated to ensure compliance with relevant regulations. As the definition above suggests, CTUs also conduct and support other types of study apart from RCTs, providing a range of methodological and subject-based expertise.

However, despite their central role in the conduct and design of trials, (and other evaluation designs) little has been written about how CTUs have integrated qualitative work within their organisation at a time when such methods are, as stated above, now recognised as an important aspect of RCTs and evaluation studies more generally. This is a significant gap, since integration at the organisational level arguably shapes how qualitative research is integrated within individual studies, and thus it is valuable to understand how CTUs have approached the task. There are different ways of involving qualitative work in trials units, such as partnering with other departments (e.g. social science) or employing qualitative researchers directly. Qualitative research can be imagined and configured in different ways—as a method that generates data to inform future trial and intervention design, as an embedded component within an RCT or other evaluation type, or as a parallel strand of research focusing on lived experiences of illness, for instance. Understanding how trials units have integrated qualitative research is valuable, as it can shed light on which strategies show promise, and in which contexts, and how qualitative research is positioned within the field of trials research, foregrounding the value of qualitative research. However, although much has been written about its use within trials, few accounts exist of how trials units have integrated qualitative research within their systems and structures.

This paper discusses the process of embedding qualitative research within the work of one CTU—Cardiff University’s Centre for Trials Research (CTR). It highlights facilitators of this process and identifies challenges to integration. We use the Normalisation Process Theory (NPT) as a framework to structure our experience and approach. The key gap addressed by this paper is the implementation of strategies to integrate qualitative research (a relatively newly adopted set of practices and processes) within CTU systems and structures. We acknowledge from the outset that there are multiple ways of approaching this task. What follows therefore is not a set of recommendations for a preferred or best way to integrate qualitative research, as this will comprise diverse actions according to specific contexts. Rather, we examine the processes through which integration occurred in our own setting and highlight the potential value of these insights for others engaged in the work of promoting qualitative research within trials units.

### Background to the integration of qualitative research within CTR

The CTR was formed in 2015 [10]. It brought together three existing trials units at Cardiff University: the South East Wales Trials Unit, the Wales Cancer Trials Unit, and the Haematology Clinical Trials Unit. From its inception, the CTR had a stated aim of developing a programme of qualitative research and integrating it within trials and other studies. In the sections below, we map these approaches onto the framework offered by Normalisation Process Theory to understand the processes through which they helped achieve embedding and integration of qualitative research.

CTR’s aims (including those relating to the development of qualitative research) were included within its strategy documents and communicated to others through infrastructure funding applications, annual reports and its website. A Qualitative Research Group (QRG), which had previously existed within the South East Wales Trials Unit, with dedicated funding for methodological specialists and group lead academics, was a key mechanism through which the development of a qualitative portfolio was put into action. Integration of qualitative research within Centre systems and processes occurred through the inclusion of qualitative research in study adoption processes and representation on committees. The CTR’s study portfolio provided a basis to track qualitative methods in new and existing studies, identify opportunities to embed qualitative methods within recently adopted studies (at the funding application stage) and to manage staff resources. Capacity building and training were an important focus of the QRG’s work, including training courses, mentoring, creation of an academic network open to university staff and practitioners working in the field of healthcare, presentations at CTR staff meetings and securing of PhD studentships. Standard operating procedures and methodological guidance on the design and conduct of qualitative research (e.g. templates for developing analysis plans) aimed to create a shared understanding of how to undertake high-quality research, and a means to monitor the implementation of rigorous approaches. As the QRG expanded its expertise it sought to develop innovative approaches, including the use of visual [11] and ethnographic methods [12].

### Understanding implementation—Normalisation Process Theory (NPT)

Normalisation Process Theory (NPT) provides a model with which to understand the implementation of new sets of practices and their normalisation within organisational settings. The term ‘normalisation’ refers to how new practices become routinised (part of the everyday work of an organisation) through embedding and integration [13, 14]. NPT defines implementation as ‘the social organisation of work’ and is concerned with the social processes that take place as new practices are introduced. *Embedding* involves ‘making practices routine elements of everyday life’ within an organisation. *Integration* takes the form of ‘sustaining embedded practices in social contexts’, and how these processes lead to the practices becoming (or not becoming) ‘normal and routine’ [14]. NPT is concerned with the factors which promote or ‘inhibit’ attempts to embed and integrate the operationalisation of new practices [13–15].

Embedding new practices is therefore achieved through implementation—which takes the form of interactions in specific contexts. Implementation is operationalised through four ‘generative mechanisms’—*coherence*, *cognitive participation*, *collective action* and *reflexive monitoring* [14]. Each mechanism is characterised by *components* comprising immediate and organisational work, with actions of individuals and organisations (or groups of individuals) interdependent. The mechanisms operate partly through forms of investment (i.e. meaning, commitment, effort, and comprehension) [14].

*Coherence* refers to how individuals/groups make sense of, and give meaning to, new practices. Sense-making concerns the coherence of a practice—whether it ‘holds together’, and its differentiation from existing activities [15]. *Communal and individual specification* involve understanding new practices and their potential benefits for oneself or an organisation. Individuals consider what new practices mean for them in terms of tasks and responsibilities (*internalisation*) [14].

NPT frames the second mechanism, *cognitive participation*, as the building of a ‘community of practice’. For a new practice to be initiated, individuals and groups within an organisation must commit to it [14, 15]. *Cognitive participation* occurs through *enrolment*—how people relate to the new practice; *legitimation*—the belief that it is right for them to be involved; and *activation*—defining which actions are necessary to sustain the practice and their involvement [14]. Making the new practices work may require changes to roles (new responsibilities, altered procedures) and reconfiguring how colleagues work together (changed relationships).

Third, *Collective Action* refers to ‘the operational work that people do to enact a set of practices’ [14]. Individuals engage with the new practices (*interactional workability*) reshaping how members of an organisation interact with each other, through creation of new roles and expectations (*relational interaction*) [15]. *Skill set workability* concerns how the work of implementing a new set of practices is distributed and the necessary roles and skillsets defined [14]. *Contextual integration* draws attention to the incorporation of a practice within social contexts, and the potential for aspects of these contexts, such as systems and procedures, to be modified as a result [15].

*Reflexive monitoring* is the final implementation mechanism. *Collective* and *individual appraisal* evaluate the value of a set of practices, which depends on the collection of information—formally and informally (*systematisation*). Appraisal may lead to *reconfiguration* in which procedures of the practice are redefined or reshaped [14, 15].

## Methods

We sought to map the following: (1) the strategies used to embed qualitative research within the Centre, (2) key facilitators, and (3) barriers to their implementation. Through focused group discussions during the monthly meetings of the CTR QRG and in discussion with the CTR senior management team throughout 2019–2020 we identified nine types of documents (22 individual documents in total) produced within the CTR which had relevant information about the integration of qualitative research within its work (Table 1). The QRG had an ‘open door’ policy to membership and welcomed all staff/students with an interest in qualitative research. It included researchers who were employed specifically to undertake qualitative research and other staff with a range of study roles, including trial managers, statisticians, and data managers. There was also diversity in terms of career stage, including PhD students, mid-career researchers and members of the Centre’s Executive team. Membership was therefore largely self-selected, and comprised of individuals with a role related to, or an interest in, embedding qualitative research within trials. However, the group brought together diverse methodological perspectives and was not solely comprised of methodological ‘champions’ whose job it was to promote the development of qualitative research within the centre. Thus whilst the group (and by extension, the authors of this paper) had a shared appreciation of the value of qualitative research within a trials centre, they also brought varied methodological perspectives and ways of engaging with it.

**Table 1:** List of documents examined to identify strategies used to integrate qualitative research

Document name/type	Number of individual documents
NISCHR Clinical Trials Unit Grant Application, 2014 (workplan covering 2015–2018)	1
Draft of CTR Qualitative Research Group Strategy Document (dated 12.04.17)	1
CTR Progress Report and Extension Bid to Health and Care Research Wales, 2017 (covering grant period 2018–2020)	1
CTR Organogram (2019)	1
A review of Standard Operating Procedures (SOPs) active in 2019 referring to qualitative research, including Qualitative Analysis Plan and Qualitative Analysis Quality Assurance	15
Draft of CTR Methodology Strategy 2020	1
CTR website, including the section on qualitative research (2020)	1
CTR brochure for new collaborators	1
**Total**	22

All members of the QRG (*n* = 26) were invited to take part in a face-to-face, day-long workshop in February 2019 on ‘How to optimise and operationalise qualitative research in trials: reflections on CTR structure’. The workshop was attended by 12 members of staff and PhD students, including members of the QRG and the CTR’s senior management team. Recruitment to the workshop was therefore inclusive, and to some extent opportunistic, but all members of the QRG were able to contribute to discussions during regular monthly group meetings and the drafting of the current paper.

The aim of the workshop was to bring together information from the documents in Table 1 to generate discussion around the key strategies (and their component activities) that had been adopted to integrate qualitative research into CTR, as well as barriers to, and facilitators of, their implementation. The agenda for the workshop involved four key areas: development and history of the CTR model; mapping the current model within CTR; discussing the structure of other CTUs; and exploring the advantages and disadvantages of the CTR model.

During the workshop, we discussed the use of NPT to conceptualise how qualitative research had been embedded within CTR’s systems and practices. The group produced spider diagrams to map strategies and actions on to the four key domains (or ‘generative mechanisms’ of NPT) summarised above, to aid the understanding of how they had functioned, and the utility of NPT as a framework. This is summarised in Table 2.

**Table 2:** Summary of the strategies and their functioning in terms of NPT generative mechanisms 13–15

Generative mechanisms	Comprises	How activities in CTR operated via these mechanisms to embed qualitative research within the centre					
		**Inclusion of qualitative research in higher level strategy and infrastructure funding**	**Formation of qualitative research team and appointment of an academic lead**	**Inclusion in centre systems—e.g. study adoption group**	**Capacity building and training**	**Methodological guidance and procedures**	**Methods development and innovation**
Coherence—sense-making	Differentiation, communal specification, individual specification and internalisation	**Differentiates** qualitative research from other approaches. **Specifies** aims and benefits for organisation and individuals	Creates group identity (**differentiation**). Establishes roles and tasks/responsibilities (**individual specification**)	**Specifies** individuals’ responsibility for conducting/supporting qualitative research	Increases understanding of qualitative work (**differentiation/internalisation**) and aims/benefits (**communal specification**). Enables individuals to specify and understand responsibilities and tasks (**internalisation**)	**Differentiates** qualitative research from other practices. **Specifies** individuals’ responsibilities/tasks	Develops understanding of benefits and aims of qualitative research (**communal specification**). Encourages individuals to consider how they can contribute to methodological development **(individual specification**)
Cognitive participation—who is involved?	Initiation, enrolment, legitimation, activation	Individual involvement **legitimised** by connecting qualitative research to centre aims	Creates organisational structure needed for individuals to contribute (**enrolment**). ‘Drives forward’ qualitative research, and establishes systems/policies/procedures (**activation**)	**Legitimises** qualitative research. Creates process and expectations around how individuals should contribute** (enrolment)**		Sets out ‘actions and procedures’ (**activation**) and how to organise tasks and relationships between people (**enrolment**)	
Collective Action—‘how the work is done'	Interactional workability, relational integration, skill set workability, contextual integration	Allocates resources for qualitative research **(contextual integration)**	Allocates work (**skill set workability**). Manages how qualitative research is conducted and provides point of contact with other teams **(interactional workability**)	Shapes how people are expected to interact with qualitative research (**interactional workability**. Inclusion in study adoption processes aids **contextual integration** through allocation of resources	Training increases staff confidence in conducting qualitative research (**relational integration**, **skillset workability**)	Guides interaction with qualitative methods and colleagues (**interactional workability**). SOPs and guidance increase accountability **(relational integration**). Protocols and policies manage practice (**contextual integration**)	
Reflexive monitoring—appraisal and reconfiguration	Systematization, communal appraisal, individual appraisal, reconfiguration	Strategy development and reporting involves review of practices and further development (**systematisation** and **reconfiguration**)	Portfolio and meetings enable monitoring of progress and identification of new opportunities (**systematisation, reconfiguration**)		Evaluation of teaching and capacity building activities enables **communal** and **individual appraisal**	Review of extent to which guidance/SOPs improve quality (**systematisation)**	**Individual** and **communal appraisal** leads to development of revised/new methods (**reconfiguration)**

Detailed notes were made during the workshop. A core writing group then used these notes and the documents in Table 1 to develop a draft of the current paper. This was circulated to all members of the CTR QRG (*n* = 26) and stored within a central repository accessible to them to allow involvement and incorporate the views of those who were not able to attend the workshop. This draft was again presented for comments in the monthly CTR QRG meeting in February 2021 attended by *n* = 10. The Standards for QUality Improvement Reporting Excellence 2.0 (SQUIRE) guidelines were used to inform the structure and content of the paper (see supplementary material) [16].

### Findings

In the following sections, we describe the strategies CTR adopted to integrate qualitative research. These are mapped against NPT’s four generative mechanisms to explore the processes through which the strategies promoted integration, and facilitators of and barriers to their implementation. A summary of the strategies and their functioning in terms of the generative mechanisms is provided in Table 2.

#### Coherence—making sense of qualitative research

In CTR, many of the actions taken to build a portfolio of qualitative research were aimed at enabling colleagues, and external actors, to make sense of this set of methodologies. Centre-level strategies and grant applications for infrastructure funding highlighted the value of qualitative research, the added benefits it would bring, and positioned it as a legitimate set of practices alongside existing methods. For example, a 2014 application for renewal of trials unit infrastructure funding stated:

We are currently in the process of undertaking […] restructuring for our qualitative research team and are planning similar for trial management next year. The aim of this restructuring is to establish greater hierarchical management and opportunities for staff development and also provide a structure that can accommodate continuing growth.

Within the CTR, various forms of communication on the development of qualitative research were designed to enable staff and students to make sense of it, and to think through its potential value for them, and ways in which they might engage with it. These included presentations at staff meetings, informal meetings between project teams and the qualitative group lead, and the visibility of qualitative research on the public-facing Centre website and Centre committees and systems. For instance, qualitative methods were included (and framed as a distinct set of practices) within study adoption forms and committee agendas. Information for colleagues described how qualitative methods could be incorporated within funding applications for RCTs and other evaluation studies to generate new insights into questions research teams were already keen to answer, such as influences on intervention implementation fidelity. Where externally based chief investigators approached the Centre to be involved in new grant applications, the existence of the qualitative team and group lead enabled the inclusion of qualitative research to be actively promoted at an early stage, and such opportunities were highlighted in the Centre’s brochure for new collaborators. Monthly qualitative research network meetings—advertised across CTR and to external research collaborators, were also designed to create a shared understanding of qualitative research methods and their utility within trials and other study types (e.g. intervention development, feasibility studies, and observational studies). Training events (discussed in more detail below) also aided sense-making.

Several factors facilitated the promotion of qualitative research as a distinctive and valuable entity. Among these was the influence of the broader methodological landscape within trial design which was promoting the value of qualitative research, such as guidance on the evaluation of complex interventions by the Medical Research Council [17], and the growing emphasis placed on process evaluations within trials (with qualitative methods important in understanding participant experience and influences on implementation) [5]. The attention given to lived experience (both through process evaluations and the move to embed public involvement in trials) helped to frame qualitative research within the Centre as something that was appropriate, legitimate, and of value. Recognition by research funders of the value of qualitative research within studies was also helpful in normalising and legitimising its adoption within grant applications.

The inclusion of qualitative methods within influential methodological guidance helped CTR researchers to develop a ‘shared language’ around these methods, and a way that a common understanding of the role of qualitative research could be generated. One barrier to such sense-making work was the varying extent to which staff and teams had existing knowledge or experience of qualitative research. This varied across methodological and subject groups within the Centre and reflected the history of the individual trials units which had merged to form the Centre.

#### Cognitive participation—legitimising qualitative research

Senior CTR leaders promoted the value and legitimacy of qualitative research. Its inclusion in centre strategies, infrastructure funding applications, and in public-facing materials (e.g. website, investigator brochures), signalled that it was appropriate for individuals to conduct qualitative research within their roles, or to support others in doing so. Legitimisation also took place through informal channels, such as senior leadership support for qualitative research methods in staff meetings and participation in QRG seminars. Continued development of the QRG (with dedicated infrastructure funding) provided a visible identity and equivalence with other methodological groups (e.g. trial managers, statisticians).

Staff were asked to engage with qualitative research in two main ways. First, there was an expansion in the number of staff for whom qualitative research formed part of their formal role and responsibilities. One of the three trials units that merged to form CTR brought with it a qualitative team comprising methodological specialists and a group lead. CTR continued the expansion of this group with the creation of new roles and an enlarged nucleus of researchers for whom qualitative research was the sole focus of their work. In part, this was linked to the successful award of projects that included a large qualitative component, and that were coordinated by CTR (see Table 3 which describes the PUMA study).

**Table 3:** Case study—the PUMA study—optimising and operationalising qualitative work in a trials unit

The ‘Paediatric early warning system: Utilisation and Mortality Avoidance’ (PUMA) study was commissioned by the UK National Institute for Health Research to develop, implement and evaluate an improvement programme to optimise the detection of and response to deterioration in hospitalised children [12]. An evidence-based, theoretically informed paediatric early warning system improvement programme was implemented in two UK general hospitals with no onsite Paediatric Intensive Care Unit (PICU) and two tertiary (onsite PICU) hospitals between November 2016 and October 2017. Quantitative data were used to assess the impact of the programme on adverse event rates in children. Qualitative methods were used to inform the development of the programme, review implementation processes, and assess system changes and associated outcomes [12]. The PUMA team consisted of a mix of paediatric consultants (*n* = 6), nurse specialists (*n* = 3), statisticians (*n* = 3), implementation scientists (*n* = 2) and medical sociologists (*n* = 5). All non-clinical members of the team were employed by the CTR, except for one: a professor of medical sociology, who was co-chief investigator. Collaborative and ongoing work on the development and implementation of the PUMA programme, and the presentation of complex mixed-methods case studies demonstrated the value of qualitative methods for informing implementation processes and interpreting quantitative data, aligning changes in event rates to changes in practice. Exposure of clinical and non-qualitative team members to qualitative methods served to legitimise the qualitative work within the study team and communicate its value to other CTR staff members. One senior clinician who was part of the team described how ‘I now appreciate some of the rigorous methodology involved and the language of qualitative research… thematic analysis, triangulation, realist evaluation, normalisation are now terms that mean something to me.’ (quotation from research team presentation)	

Members of the QRG were encouraged to develop their own research ideas and to gain experience as principal investigators, and group seminars were used to explore new ideas and provide peer support. This was communicated through line management, appraisal, and informal peer interaction. Boundaries were not strictly demarcated (i.e. staff located outside the qualitative team were already using qualitative methods), but the new team became a central focus for developing a growing programme of work.

Second, individuals and studies were called upon to engage in new ways with qualitative research, and with the qualitative team. A key goal for the Centre was that groups developing new research ideas should give more consideration in general to the potential value and inclusion of qualitative research within their funding applications. Specifically, they were asked to do this by thinking about qualitative research at an early point in their application’s development (rather than ‘bolting it on’ after other elements had been designed) and to draw upon the expertise and input of the qualitative team. An example was the inclusion of questions on qualitative methods within the Centre’s study adoption form and representation from the qualitative team at the committee which reviewed new adoption requests. Where adoption requests indicated the inclusion of qualitative methods, colleagues were encouraged to liaise with the qualitative team, facilitating the integration of its expertise from an early stage. Qualitative seminars offered an informal and supportive space in which researchers could share initial ideas and refine their methodological approach. The benefits of this included the provision of sufficient time for methodological specialists to be involved in the design of the proposed qualitative component and ensuring adequate costings had been drawn up. At study adoption group meetings, scrutiny of new proposals included consideration of whether new research proposals might be strengthened through the use of qualitative methods where these had not initially been included. Meetings of the QRG—which reviewed the Centre’s portfolio of new studies and gathered intelligence on new ideas—also helped to identify, early on, opportunities to integrate qualitative methods. Communication across teams was useful in identifying new research ideas and embedding qualitative researchers within emerging study development groups.

Actions to promote greater use of qualitative methods in funding applications fed through into a growing number of studies with a qualitative component. This helped to increase the visibility and legitimacy of qualitative methods within the Centre. For example, the PUMA study [12], which brought together a large multidisciplinary team to develop and evaluate a Paediatric early warning system, drew heavily on qualitative methods, with the qualitative research located within the QRG. The project introduced an extensive network of collaborators and clinical colleagues to qualitative methods and how they could be used during intervention development and the generation of case studies. Further information about the PUMA study is provided in Table 3.

Increasing the legitimacy of qualitative work across an extensive network of staff, students and collaborators was a complex process. Set within the continuing dominance of quantitative methods with clinical trials, there were variations in the extent to which clinicians and other collaborators embraced the value of qualitative methods. Research funding schemes, which often continued to emphasise the quantitative element of randomised controlled trials, inevitably fed through into the focus of new research proposals. Staff and external collaborators were sometimes uncertain about the added value that qualitative methods would bring to their trials. Across the CTR there were variations in the speed at which qualitative research methods gained legitimacy, partly based on disciplinary traditions and their influences. For instance, population health trials, often located within non-health settings such as schools or community settings, frequently involved collaboration with social scientists who brought with them experience in qualitative methods. Methodological guidance in this field, such as MRC guidance on process evaluations, highlighted the value of qualitative methods and alternatives to the positivist paradigm, such as the value of realist RCTs. In other, more clinical areas, positivist paradigms had greater dominance. Established practices and methodological traditions across different funders also influenced the ease of obtaining funding to include qualitative research within studies. For drugs trials (CTIMPs), the influence of regulatory frameworks on study design, data collection and the allocation of staff resources may have played a role. Over time, teams gained repeated experience of embedding qualitative research (and researchers) within their work and took this learning with them to subsequent studies. For example, the senior clinician quoted within the PUMA case study (Table 3 below) described how they had gained an appreciation of the rigour of qualitative research and an understanding of its language. Through these repeated interactions, embedding of qualitative research within studies started to become the norm rather than the exception.

#### Collective action—operationalising qualitative research

*Collective action* concerns the operationalisation of new practices within organisations—the allocation and management of the work, how individuals interact with each other, and the work itself. In CTR the formation of a Qualitative Research Group helped to allocate and organise the work of building a portfolio of studies. Researchers across the Centre were called upon to interact with qualitative research in new ways. Presentations at staff meetings and the inclusion of qualitative research methods in portfolio study adoption forms were examples of this (*interactive workability*). It was operationalised by encouraging study teams to liaise with the qualitative research lead. Development of standard operating procedures, templates for costing qualitative research and methodological guidance (e.g. on analysis plans) also helped encourage researchers to interact with these methods in new ways. For some qualitative researchers who had been trained in the social sciences, working within a trials unit meant that they needed to interact in new and sometimes unfamiliar ways with standard operating procedures, risk assessments, and other trial-based systems. Thus, training needs and capacity-building efforts were multidirectional.

Whereas there had been a tendency for qualitative research to be ‘bolted on’ to proposals for RCTs, the systems described above were designed to embed thinking about the value and design of the qualitative component from the outset. They were also intended to integrate members of the qualitative team with trial teams from an early stage to promote effective integration of qualitative methods within larger trials and build relationships over time.

Standard Operating Procedures (SOPs), formal and informal training, and interaction between the qualitative team and other researchers increased the *relational workability* of qualitative methods within the Centre—the confidence individuals felt in including these methods within their studies, and their accountability for doing so. For instance, study adoption forms prompted researchers to interact routinely with the qualitative team at an early stage, whilst guidance on costing grants provided clear expectations about the resources needed to deliver a proposed set of qualitative data collection.

Formation of the Qualitative Research Group—comprised of methodological specialists, created new roles and skillsets (*skill set workability*). Research teams were encouraged to draw on these when writing funding applications for projects that included a qualitative component. Capacity-building initiatives were used to increase the number of researchers with the skills needed to undertake qualitative research, and for these individuals to develop their expertise over time. This was achieved through formal training courses, academic seminars, mentoring from experienced colleagues, and informal knowledge exchange. Links with external collaborators and centres engaged in building qualitative research supported these efforts. Within the Centre, the co-location of qualitative researchers with other methodological and trial teams facilitated knowledge exchange and building of collaborative relationships, whilst grouping of the qualitative team within a dedicated office space supported a collective identity and opportunities for informal peer support.

Some aspects of the context in which qualitative research was being developed created challenges to operationalisation. Dependence on project grants to fund qualitative methodologists meant that there was a continuing need to write further grant applications whilst limiting the amount of time available to do so. Similarly, researchers within the team whose role was funded largely by specific research projects could sometimes find it hard to create sufficient time to develop their personal methodological interests. However, the cultivation of a methodologically varied portfolio of work enabled members of the team to build significant expertise in different approaches (e.g. ethnography, discourse analysis) that connected individual studies.

#### Reflexive monitoring—evaluating the impact of qualitative research

Inclusion of questions/fields relating to qualitative research within the Centre’s study portfolio database was a key way in which information was collected (*systematisation*). It captured numbers of funding applications and funded studies, research design, and income generation. Alongside this database, a qualitative resource planner spreadsheet was used to link individual members of the qualitative team with projects and facilitate resource planning, further reinforcing the core responsibilities and roles of qualitative researchers within CTR. As with all staff in the Centre, members of the qualitative team were placed on ongoing rather than fixed-term contracts, reflecting their core role within CTR. Planning and strategy meetings used the database and resource planner to assess the integration of qualitative research within Centre research, identify opportunities for increasing involvement, and manage staff recruitment and sustainability of researcher posts. Academic meetings and day-to-day interaction fulfilled informal appraisal of the development of the group, and its position within the Centre. Individual appraisal was also important, with members of the qualitative team given opportunities to shape their role, reflect on progress, identify training needs, and further develop their skillset, particularly through line management systems.

These forms of *systematisation* and *appraisal* were used to reconfigure the development of qualitative research and its integration within the Centre. For example, group strategies considered how to achieve long-term integration of qualitative research from its initial embedding through further promoting the belief that it formed a core part of the Centre’s business. The visibility and legitimacy of qualitative research were promoted through initiatives such as greater prominence on the Centre’s website. Ongoing review of the qualitative portfolio and discussion at academic meetings enabled the identification of areas where increased capacity would be helpful, both for qualitative staff, and more broadly within the Centre. This prompted the qualitative group to develop an introductory course to qualitative methods open to all Centre staff and PhD students, aimed at increasing understanding and awareness. As the qualitative team built its expertise and experience it also sought to develop new and innovative approaches to conducting qualitative research. This included the use of visual and diary-based methods [11] and the adoption of ethnography to evaluate system-level clinical interventions [12]. Restrictions on conventional face-to-face qualitative data collection due to the COVID-19 pandemic prompted rapid adoption of virtual/online methods for interviews, observation, and use of new internet platforms such as Padlet—a form of digital note board.

## Discussion

In this paper, we have described the work undertaken by one CTU to integrate qualitative research within its studies and organisational culture. The parallel efforts of many trials units to achieve these goals arguably come at an opportune time. The traditional designs of RCTs have been challenged and re-imagined by the increasing influence of realist evaluation [6, 18] and the widespread acceptance that trials need to understand implementation and intervention theory as well as assess outcomes [17]. Hence the widespread adoption of embedded mixed methods process evaluations within RCTs. These broad shifts in methodological orthodoxies, the production of high-profile methodological guidance, and the expectations of research funders all create fertile ground for the continued expansion of qualitative methods within trials units. However, whilst much has been written about the importance of developing qualitative research and the possible approaches to integrating qualitative and quantitative methods within studies, much less has been published on how to operationalise this within trials units. Filling this lacuna is important. Our paper highlights how the integration of a new set of practices within an organisation can become embedded as part of its ‘normal’ everyday work whilst also shaping the practices being integrated. In the case of CTR, it could be argued that the integration of qualitative research helped shape how this work was done (e.g. systems to assess progress and innovation).

In our trials unit, the presence of a dedicated research group of methodological specialists was a key action that helped realise the development of a portfolio of qualitative research and was perhaps the most visible evidence of a commitment to do so. However, our experience demonstrates that to fully realise the goal of developing qualitative research, much work focuses on the interaction between this ‘new’ set of methods and the organisation into which it is introduced. Whilst the team of methodological specialists was tasked with, and ‘able’ to do the work, the ‘work’ itself needed to be integrated and embedded within the existing system. Thus, alongside the creation of a team and methodological capacity, promoting the legitimacy of qualitative research was important to communicate to others that it was both a distinctive and different entity, yet similar and equivalent to more established groups and practices (e.g. trial management, statistics, data management). The framing of qualitative research within strategies, the messages given out by senior leaders (formally and informally) and the general visibility of qualitative research within the system all helped to achieve this.

Normalisation Process Theory draws our attention to the concepts of *embedding* (making a new practice routine, normal within an organisation) and *integration*—the long-term sustaining of these processes. An important process through which embedding took place in our centre concerned the creation of messages and systems that called upon individuals and research teams to interact with qualitative research. Research teams were encouraged to think about qualitative research and consider its potential value for their studies. Critically, they were asked to do so at specific points, and in particular ways. Early consideration of qualitative methods to maximise and optimise their inclusion within studies was emphasised, with timely input from the qualitative team. Study adoption systems, centre-level processes for managing financial and human resources, creation of a qualitative resource planner, and awareness raising among staff, helped to reinforce this. These processes of embedding and integration were complex and they varied in intensity and speed across different areas of the Centre’s work. In part this depended on existing research traditions, the extent of prior experience of working with qualitative researchers and methods, and the priorities of subject areas and funders. Centre-wide systems, sometimes linked to CTR’s operation as a CTU, also helped to legitimise and embed qualitative research, lending it equivalence with other research activity. For example, like all CTUs, CTR was required to conform with the principles of Good Clinical Practice, necessitating the creation of a quality management system, operationalised through standard operating procedures for all areas of its work. Qualitative research was included, and became embedded, within these systems, with SOPs produced to guide activities such as qualitative analysis.

NPT provides a helpful way of understanding how trials units might integrate qualitative research within their work. It highlights how new practices interact with existing organisational systems and the work needed to promote effective interaction. That is, alongside the creation of a team or programme of qualitative research, much of the work concerns how members of an organisation understand it, engage with it, and create systems to sustain it. Embedding a new set of practices may be just as important as the quality or characteristics of the practices themselves. High-quality qualitative research is of little value if it is not recognised and drawn upon within new studies for instance. NPT also offers a helpful lens with which to understand *how* integration and embedding occur, and the mechanisms through which they operate. For example, promoting the legitimacy of a new set of practices, or creating systems that embed it, can help sustain these practices by creating an organisational ambition and encouraging (or requiring) individuals to interact with them in certain ways, redefining their roles accordingly. NPT highlights the ways in which integration of new practices involves bi-directional exchanges with the organisation’s existing practices, with each having the potential to re-shape the other as interaction takes place. For instance, in CTR, qualitative researchers needed to integrate and apply their methods within the quality management and other systems of a CTU, such as the formalisation of key processes within standard operating procedures, something less likely to occur outside trials units. Equally, project teams (including those led by externally based chief investigators) increased the integration of qualitative methods within their overall study design, providing opportunities for new insights on intervention theory, implementation and the experiences of practitioners and participants.

We note two aspects of the normalisation processes within CTR that are slightly less well conceptualised by NPT. The first concerns the emphasis within *coherence* on identifying the distinctiveness of new practices, and how they differ from existing activities. Whilst differentiation was an important aspect of the integration of qualitative research in CTR, such integration could be seen as operating partly through processes of de-differentiation, or at least equivalence. That is, part of the integration of qualitative research was to see it as similar in terms of rigour, coherence, and importance to other forms of research within the Centre. To be viewed as similar, or at least comparable to existing practices, was to be legitimised.

Second, whilst NPT focuses mainly on the interaction between a new set of practices and the organisational context into which it is introduced, our own experience of introducing qualitative research into a trials unit was shaped by broader organisational and methodological contexts. For example, the increasing emphasis placed upon understanding implementation processes and the experiences of research participants in the field of clinical trials (e.g. by funders), created an environment conducive to the development of qualitative research methods within our Centre. Attempts to integrate qualitative research within studies were also cross-organisational, given that many of the studies managed within the CTR drew together multi-institutional teams. This provided important opportunities to integrate qualitative research within a portfolio of studies that extended beyond CTR and build a network of collaborators who increasingly included qualitative methods within their funding proposals. The work of growing and integrating qualitative research within a trials unit is an ongoing one in which ever-shifting macro-level influences can help or hinder, and where the organisations within which we work are never static in terms of barriers and facilitators.

## Conclusions

The importance of utilising qualitative methods within RCTs is now widely recognised. Increased emphasis on the evaluation of complex interventions, the influence of realist methods directing greater attention to complexity and the widespread adoption of mixed methods process evaluations are key drivers of this shift. The inclusion of qualitative methods within individual trials is important and previous research has explored approaches to their incorporation and some of the challenges encountered. Our paper highlights that the integration of qualitative methods at the organisational level of the CTU can shape how they are taken up by individual trials. Within CTR, it can be argued that qualitative research achieved high levels of integration, as conceptualised by Normalisation Process Theory. Thus, qualitative research became recognised as a coherent and valuable set of practices, secured legitimisation as an appropriate focus of individual and organisational activity and benefitted from forms of *collective action* which operationalised these organisational processes. Crucially, the *routinisation* of qualitative research appeared to be sustained, something which NPT suggests helps define integration (as opposed to initial embedding). However, our analysis suggested that the degree of integration varied by trial area. This variation reflected a complex mix of factors including disciplinary traditions, methodological guidance, existing (un)familiarity with qualitative research, and the influence of regulatory frameworks for certain clinical trials.

NPT provides a valuable framework with which to understand how these processes of embedding and integration occur. Our use of NPT draws attention to the importance of sense-making and legitimisation as important steps in introducing a new set of practices within the work of an organisation. Integration also depends, across each mechanism of NPT, on the building of effective relationships, which allow individuals and teams to work together in new ways. By reflecting on our experiences and the decisions taken within CTR we have made explicit one such process for embedding qualitative research within a trials unit, whilst acknowledging that approaches may differ across trials units. Mindful of this fact, and the focus of the current paper on one trials unit’s experience, we do not propose a set of recommendations for others who are working to achieve similar goals. Rather, we offer three overarching reflections (framed by NPT) which may act as a useful starting point for trials units (and other infrastructures) seeking to promote the adoption of qualitative research.

First, whilst research organisations such as trials units are highly heterogenous, processes of embedding and integration, which we have foregrounded in this paper, are likely to be important across different contexts in sustaining the use of qualitative research. Second, developing a plan for the integration of qualitative research will benefit from mapping out the characteristics of the extant system. For example, it is valuable to know how familiar staff are with qualitative research and any variations across teams within an organisation. Thirdly, NPT frames integration as a process of implementation which operates through key generative mechanisms—*coherence*, *cognitive participation*, *collective action* and *reflexive monitoring*. These mechanisms can help guide understanding of which actions help achieve embedding and integration. Importantly, they span multiple aspects of how organisations, and the individuals within them, work. The ways in which people make sense of a new set of practices (*coherence*), their commitment towards it (*cognitive participation*), how it is operationalised (*collective action*) and the evaluation of its introduction (*reflexive monitoring*) are all important. Thus, for example, qualitative research, even when well organised and operationalised within an organisation, is unlikely to be sustained if appreciation of its value is limited, or people are not committed to it.

We present our experience of engaging with the processes described above to open dialogue with other trials units on ways to operationalise and optimise qualitative research in trials. Understanding how best to integrate qualitative research within these settings may help to fully realise the significant contribution which it makes the design and conduct of trials.

### Supplementary Information

**Supplementary Material 1.**

## Data Availability

Some documents cited in this paper are either freely available from the Centre for Trials Research website or can be requested from the author for correspondence.
