# Supplementary material for: Integrating qualitative research within a clinical trials unit: developing strategies and understanding their implementation in contexts
Source: Trials. 2024 May 16;25:323. doi: 10.1186/s13063-024-08124-7 (PMC11097452; doi:10.1186/s13063-024-08124-7)
Supplement: Supplementary file 1 — Supplementary Material 1. [file 13063_2024_8124_MOESM1_ESM.docx]

**Revised Standards for Quality Improvement Reporting Excellence - SQUIRE 2.0**

| **Title and Abstract** | |
| --- | --- |
| Title | Integrating qualitative research within a clinical trials unit: developing strategies and understanding their implementation in contexts |
| Abstract | Background/Aims: The value of using qualitative methods within clinical trials is widely recognised. How qualitative research is integrated within trials units to achieve this is less clear. This paper describes the process through which qualitative research has been integrated within Cardiff University’s Centre for Trials Research (CTR) in Wales, UK. We highlight facilitators of, and challenges to, integration.  Methods: We held group discussions on the work of the Qualitative Research Group (QRG) within CTR. The content of these discussions, materials for a presentation in CTR, and documents relating to the development of the QRG were interpreted at a workshop attended by group members. Normalisation Process Theory (NPT) was used to structure analysis. A writing group prepared a document for input from members of CTR, forming the basis of this paper.  Results: Actions to integrate qualitative research comprised: its inclusion in Centre strategies; formation of a QRG with dedicated funding/roles; embedding of qualitative research within operating systems; capacity building/training; monitoring opportunities to include qualitative methods in studies; maximising the quality of qualitative research and developing methodological innovation. Facilitators of these actions included: the influence of the broader methodological landscape within trial/study design and its promotion of the value of qualitative research; and close physical proximity of CTR qualitative staff/students allowing sharing of methodological approaches. Introduction of innovative qualitative methods generated interest among other staff groups. Challenges included: pressure to under-resource qualitative components of research; preference for a statistical stance historically in some research areas and funding structures; and difficulties faced by qualitative researchers carving out individual academic profiles when working across trials/studies.  Conclusions: Given that CTUs are pivotal to the design and conduct of RCTs and related study types across multiple disciplines, integrating qualitative research into Trials Units is crucial if its role is to be fully realised. We have made explicit one trials unit’s experience of embedding qualitative research and present this to open dialogue on ways to operationalise and optimise qualitative research in trials. NPT provides a valuable framework with which to theorise these processes, including the importance of sense-making and legitimisation when introducing new practices within organisations. |
| **Introduction** | |
| Problem description | Despite their central role in the conduct and design of trials, (and other evaluation designs) little has been written about how CTUs have integrated qualitative work within their organisation. Few accounts exist of how trials units have integrated qualitative research within their systems and structures. |
| Available knowledge | The value of using qualitative methods within Randomised Control Trials (RCTs) is widely recognised. Qualitative research generates important evidence on factors affecting trial recruitment/retention and implementation, aiding interpretation of quantitative data. A high proportion of trials are based within or involve clinical trials units (CTUs). |
| Rationale | The absence of work on how qualitative research has been integrated within CTUs is a significant gap. Integration at the organisational level may shape how qualitative research is integrated within individual studies, and thus it is valuable to understand how CTUs have approached the task.  Understanding how trials units have integrated qualitative research is valuable, as it can shed light on which strategies show promise, and in which contexts, and how qualitative research is positioned within the field of trials research, foregrounding the value of qualitative research. |
| Specific aims | This paper describes the process through which qualitative research has been integrated within Cardiff University’s Centre for Trials Research (CTR) in Wales, UK. We highlight facilitators of, and challenges to, integration. We map out: (1) the strategies used to embed qualitative research within the Centre; (2) key facilitators; and (3) barriers to their implementation |
| **Methods** | |
| Context | The Centre for Trials Research (CTR) was formed in 2015. It brought together three existing trials units at Cardiff University: the South East Wales Trials Unit, the Wales Cancer Trials Unit, and the Haematology Clinical Trials Unit. From its inception, the CTR had a stated aim of developing a programme of qualitative research and integrating it within trials and other studies. |
| Intervention(s) | The paper is not concerned with a discrete intervention. It discusses the process of embedding qualitative research within the work of one CTU – Cardiff University’s Centre for Trials Research (CTR). Its focus is therefore on how new practices (qualitative research) are integrated and embedded within organisations (clinical trials units). |
| Study of the intervention(s) | Methods were:   - focused group discussions during the monthly meetings of the CTR QRG and in discussion with the CTR senior management team throughout 2019-2020 - analysis of nine types of documents (22 individual documents in total) produced within the CTR which had relevant information about the integration of qualitative research within its work - A day workshop attended by members of the QRG which brought together information from the documents analysed to generate discussion around the key strategies (and their component activities) that had been adopted to integrate qualitative research into CTR, as well as barriers to, and facilitators of, their implementation. The agenda for the workshop involved four key areas: development and history of the CTR model; mapping the current model within CTR; discussing the structure of other CTUs; exploring the advantages and disadvantages of the CTR model. |
| Measures | N/A |
| Analysis | - Documents were subjected to analysis to identify key themes. - Detailed notes were taken at the QRG workshop, including mapping out strategies and actions. - Normalisation Process Theory (NPT) was used as a theoretical framework. |
| Ethical considerations | Ethical approval was not sought as no personal or identifiable data was collected. |
| **Results** | |
| Results | Actions to integrate qualitative research comprised: its inclusion in Centre strategies; formation of a QRG with dedicated funding/roles; embedding of qualitative research within operating systems; capacity building/training; monitoring opportunities to include qualitative methods in studies; maximising the quality of qualitative research and developing methodological innovation. Facilitators of these actions included: the influence of the broader methodological landscape within trial/study design and its promotion of the value of qualitative research; and close physical proximity of CTR qualitative staff/students allowing sharing of methodological approaches. Introduction of innovative qualitative methods generated interest among other staff groups. Challenges included: pressure to under-resource qualitative components of research, preference for a statistical stance historically in some research areas and funding structures, and difficulties faced by qualitative researchers carving out individual academic profiles when working across trials/studies. |
| **Discussion** | |
| Summary | Our paper highlights how the integration of a new set of practices within an organisation can become embedded as part of its ‘normal’ everyday work, whilst also shaping the practices being integrated. In the case of CTR, the integration of qualitative research helped shape how this work was done (e.g., systems to assess progress and innovation). |
| Interpretation | N/A |
| Limitations | The paper describes the experience of a single trials unit. We acknowledge that the approaches adopted to embed qualitative research will vary across units. |
| Conclusions | Our paper highlights that the integration of qualitative methods at the organisational level of the CTU can shape how they are taken up within individual trials. Within CTR, it can be argued that qualitative research achieved high levels of integration, as conceptualised by Normalisation Process Theory.  NPT provides a valuable framework with which to understand how these processes of embedding and integration occur. Our use of NPT draws attention to the importance of sense making and legitimisation as important steps when introducing a new set of practices within the work of an organisation. Integration also depends, across each mechanism of NPT, on the building of effective relationships, which allow individuals and teams to work together in new ways. Key reflection points for other researchers concern: 1) the transferability of the importance of embedding and integration across different contexts; 2) the importance of considering characteristics of the extant system when embedding new practices; and 3) the value of considering key NPT mechanisms together to maximise the likelihood of integration being achieved.  By reflecting on our experiences and the decisions made within CTR we have made explicit one such process for embedding qualitative research within a Trials Unit |
| **Other information** | |
| Funding | No external funding was received. CTR supported authors through enabling them to allocate time for paper development and writing. |
|  |  |
